# Association of Pre-diagnostic Antibody Responses to Escherichia coli and Bacteroides fragilis Toxin Proteins with Colorectal Cancer in a European Cohort

**DOI:** 10.1080/19490976.2021.1903825

**Published:** 2021-04-20

**Authors:** Julia Butt, Mazda Jenab, Jill Werner, Veronika Fedirko, Elisabete Weiderpass, Christina C. Dahm, Anne Tjønneland, Anja Olsen, Marie-Christine Boutron-Ruault, Joseph A. Rothwell, Gianluca Severi, Rudolf Kaaks, Renée Turzanski-Fortner, Krasimira Aleksandrova, Matthias Schulze, Domenico Palli, Valeria Pala, Salvatore Panico, Rosario Tumino, Carlotta Sacerdote, Bas Bueno-de-Mesquita, Carla H. Van Gils, Inger Torhild Gram, Marko Lukic, Núria Sala, María José Sánchez Pérez, Eva Ardanaz, María-Dolores Chirlaque, Richard Palmquist, Thyra Löwenmark, Ruth C Travis, Alicia Heath, Amanda J Cross, Heinz Freisling, Semi Zouiouich, Elom Aglago, Tim Waterboer, David J. Hughes

**Affiliations:** a Infections and Cancer Epidemiology, German Cancer Research Center (DKFZ), Heidelberg, Germany; b International Agency for Research on Cancer (IARC-WHO), Lyon, France; c Department of Epidemiology, Rollins School of Public Health, Emory University, Atlanta, GA, USA; d Department of Public Health, Aarhus University, Aarhus, Denmark; e Exposome and Heredity Team, CESP (Centre de Recherche en Epidemiologie et Santé des Populations), Diet, Genes and Environment, Nutrition and Biomarkers (NAB), Danish Cancer Society Research Center, Copenhagen, Denmark; f Department of Public Health, University of Copenhagen, Denmark; g Department of Public Health, University of Aarhus, Denmark; h Cesp (Umr1018), Médecine Université Paris-Saclay, Inserm, Gustave Roussy, Villejuif, France; i Department of Statistics, Computer Science and Applications (DISIA), University of Florence, Italy; j Division of Cancer Epidemiology, German Cancer Research Center (DKFZ), Heidelberg, Germany; k Department of Nutrition and Gerontology, German Institute of Human Nutrition Potsdam-Rehbruecke, Germany; l Department of Molecular Epidemiology, German Institute of Human Nutrition, Potsdam-Rehbruecke, Nuthetal, Germany; m Institute of Nutritional Science, University of Potsdam, Nuthetal, Germany; n Institute for Cancer Research, Prevention and Clinical Network - ISPRO, Florence, Italy; o Epidemiology and Prevention Unit, Fondazione IRCCS Istituto Nazionale Dei Tumori, Milan, Italy; p Dipartimento Di Medicina Clinica E Chirurgia, Federico II University, Naples, Italy; q Cancer Registry and Histopathology Department, Provincial Health Authority (ASP 7), Ragusa, Italy; r Unit of Cancer Epidemiology, Città Della Salute E Della Scienza University-Hospital, Turin, Italy; s Former Senior Scientist, Dept. For Determinants of Chronic Diseases (DCD), National Institute for Public Health and the Environment (RIVM), Bilthoven, The Netherlands; t Former Associate Professor, Department of Gastroenterology and Hepatology, University Medical Centre, Utrecht, The Netherlands; u Former Visiting Professor, Dept. Of Epidemiology and Biostatistics, The School of Public Health, Imperial College London, London, UK; v Former Academic Icon/visiting Professor, Dept. Of Social & Preventive Medicine, Faculty of Medicine, University of Malaya, Kuala Lumpur, Malaysia; w Julius Centre for Health Sciences and Primary Care, University Medical Centre Utrecht, Utrecht University, Utrecht, The Netherlands; x Department of Community Medicine, University of Tromsø, the Arctic University of Norway, Tromsø, Norway; y Department of Community Medicine, UiT, The Arctic University of Norway, Tromsø, Norway; z Unit of Nutrition, Environment and Cancer, Cancer Epidemiology Research Program and Translational Research Laboratory, Catalan Institute of Oncology (ICO), Biomedical Research Institute (IDIBELL), Barcelona, Spain; aa Escuela Andaluza De Salud Pública (EASP), Granada, Spain; bb Instituto De Investigación Biosanitaria ibs.GRANADA, Granada, Spain; cc Centro De Investigación Biomédica En Red De Epidemiología Y Salud Pública (CIBERESP), Madrid, Spain; dd Department of Preventive Medicine and Public Health, University of Granada, Granada, Spain; ee Navarra Public Health Institute, Pamplona, Spain; ff IdiSNA, Navarra Institute for Health Research, Pamplona, Spain; gg CIBER Epidemiology and Public Health CIBERESP, Madrid, Spain; hh Department of Epidemiology, Regional Health Council, IMIB-Arrixaca, Murcia University, Murcia, Spain; ii CIBER in Epidemiology and Public Health (CIBERESP), Madrid, Spain; jj Department of Medical Biosciences, Pathology, Umeå University, Ireland; kk Cancer Epidemiology Unit, Nuffield Department of Population Health, University of Oxford, Oxford, UK; ll Department of Epidemiology and Biostatistics, School of Public Health, Imperial College London, London, UK; mm Cancer Biology and Therapeutics Group, School of Biomolecular and Biomedical Science, UCD Conway Institute, University College Dublin, Dublin, Ireland

**Keywords:** Colorectal cancer, Escherichia coli, bacteroides fragilis, serology, prospective

## Abstract

Experimental evidence has implicated genotoxic *Escherichia coli* (*E. coli*) and enterotoxigenic *Bacteroides fragilis* (ETBF) in the development of colorectal cancer (CRC). However, evidence from epidemiological studies is sparse. We therefore assessed the association of serological markers of *E. coli* and ETBF exposure with odds of developing CRC in the European Prospective Investigation into Nutrition and Cancer (EPIC) study.

Serum samples of incident CRC cases and matched controls (n = 442 pairs) were analyzed for immunoglobulin (Ig) A and G antibody responses to seven *E. coli* proteins and two isoforms of the ETBF toxin via multiplex serology. Multivariable-adjusted conditional logistic regression analyses were used to estimate odds ratios (ORs) and 95% confidence intervals (CIs) for the association of sero-positivity to *E. coli* and ETBF with CRC.

The IgA-positivity of any of the tested *E. coli* antigens was associated with higher odds of developing CRC (OR: 1.42; 95% CI: 1.05–1.91). Dual-positivity for both IgA and IgG to *E. coli* and ETBF was associated with >1.7-fold higher odds of developing CRC, with a significant association only for IgG (OR: 1.75; 95% CI: 1.04, 2.94). This association was more pronounced when restricted to the proximal colon cancers (OR: 2.62; 95% CI: 1.09, 6.29) compared to those of the distal colon (OR: 1.24; 95% CI: 0.51, 3.00) (*p_heterogeneity_ *= 0.095). Sero-positivity to *E. coli* and ETBF was associated with CRC development, suggesting that co-infection of these bacterial species may contribute to colorectal carcinogenesis. These findings warrant further exploration in larger prospective studies and within different population groups.

## Introduction

Colorectal cancer (CRC) is among the top most common cancer types with more than 1.8 million newly diagnosed cases and 880,000 deaths worldwide in 2018.^1^ Inflammation is thought to be a major mechanistic process underlying CRC development from etiological risk factors and is a possible mechanism through which bacterial infections might contribute to carcinogenesis.^2^ Indeed, microbiome dysbiosis is becoming increasingly implicated in disease pathogenesis, and some distinct bacterial species have been investigated as potential causative agents in CRC development including genotoxic and enterotoxigenic strains of *Escherichia coli* (*E. coli*) and *Bacteroides fragilis* (*B. fragilis*).^3^

Experimental and animal models support a mechanistic basis for a potential association of these bacterial species with CRC. Infection with the enterotoxigenic *B. fragilis* (ETBF) expressing the *B. fragilis* toxin (BFT) was found to promote tumorigenesis in CRC mouse models.^4–6^ BFT was shown to exert its pro-carcinogenic effects by directly damaging DNA and by inducing cell proliferation in colon epithelial cells by cleaving E-cadherin and inducing the Wnt/β-catenin pathway.^7,8^ Similar to ETBF, the enterotoxigenic *E. coli* strain harboring the polyketide synthesis (*pks)* genomic island (pks+ *E. coli*) also promoted tumorigenesis in CRC mouse models.^9,10^ The pks+ *E. coli* strain secretes colibactin, a molecule that was shown to alkylate and induce DNA double-strand breaks in cell culture, potentially inducing genomic alterations in the infected cells.^7,11,12^

Interestingly, both bacterial species have been identified in tumor tissue of CRC patients.^13–17^ A study of tumors from 88 CRC patients reported that while *E. coli* colonization associated with the microsatellite instability (MSI) CRC phenotype, colibactin-producing strains were enriched in microsatellite stable (MSS) CRC.^18^ In cell-line models, these investigators further showed that the pks+ve *E. coli* could inhibit the MLH1 mismatch repair protein, further supporting the potential promotion of genomic instability by colibactin.^18^ A pks+ *E. coli* specific mutational signature within a subset of human CRC genomes has recently been described in two independent cohorts,^19^ supportive of a genotoxic effect of pks+ *E. coli* also in human colorectal carcinogenesis. These colibactin signatures have also been identified in colorectal polyposis patients with specific *APC* splice variants.^20^ ETBF presence in CRC tumors has been significantly associated with the colonic subsite and later disease stages.^16^ Furthermore, a study of familial adenomatous polyposis (FAP) patients has reported the presence of biofilms consisting of both pks+ *E. coli* and ETBF adhering to the colonic mucosa of these patients.^21^ Such biofilms, which comprise a higher order structure of bacterial organization, have been identified in other previous studies of CRC tissue, predominantly of proximal tumor location, and were associated with inflammation and epithelial cell proliferation.^22,23^

In summary, the accumulated experimental research suggests a role for certain strains of *E. coli* and *B. fragilis* in CRC development; however, evidence from epidemiological studies is sparse.^24^ In the present study, we therefore aimed to assess whether antibody responses to *E. coli* proteins, specifically involved in biofilm formation and colibactin secretion, and the *B. fragilis* toxin are associated with higher odds of developing CRC in a prospective nested case–control study within the European Prospective Investigation into Cancer and Nutrition (EPIC) cohort. Due to the evidence for aggregation of the two species in biofilms, we hypothesized that antibody responses to proteins of both bacteria combined are associated with CRC and that this association is predominantly found for tumors in the proximal colon considering previous data on biofilm distribution in the colon.^22,23^

## Methods

### Study population

The present study is nested within the EPIC cohort, the main goal of which is to investigate the association between diet, lifestyle, environmental and genetic factors and development of cancer and other major chronic diseases.^25^ For the overall cohort, approximately 520,000 individuals, aged 35 to 70 y, were enrolled from 10 Western European countries between 1992 and 2000. The present CRC nested case–control study is based on participant data from six of these countries (France, Italy, Spain, United Kingdom, The Netherlands, and Germany). Dietary and lifestyle data, as well as serum samples, were collected at baseline, with standardized blood collection and processing protocols across the study centers. Serum samples were stored at the International Agency for Research on Cancer (IARC, Lyon, France) at −196°C and shipped on dry ice to the German Cancer Research Center (DKFZ), Heidelberg, Germany. The study was approved by the IARC ethics committee and the ethics committees of all local participating centers. Written informed consent was obtained from all study participants. The study design methods were performed in accordance with the STROBE (Strengthening the Reporting of Observational Studies in Epidemiology) guidelines [https://www.strobe-statement.org/index.php?id=strobe-home].

The design of this nested CRC case–control study has been published elsewhere.^26,27^ Briefly, pre-diagnostic serum samples from 492 CRC cases (primary tumors coded C18-C20 according to the 10^th^ revision of the International Statistical Classification of Diseases, Injury and Causes of Death, diagnosed between 1993 and 2008) with sufficient available volume of serum for the planned analyses were included. Controls (1:1) were selected by incidence density sampling from all cohort members alive and free of cancer at the time of diagnosis of the case and matched by age at blood collection (±6 months to ±2 y), sex, study center, time of the day at blood collection (±2 to 4 h interval), fasting status at blood collection (<3/3-6 h); among women, controls were also matched to cases by menopausal status (premenopausal, perimenopausal, postmenopausal, or surgically menopausal), current use of exogenous hormones (oral contraceptives or hormone therapy, yes/no), and phase of menstrual cycle at blood collection. Forty matched case–control pairs were excluded from the analysis due to low serum volume or technical errors during multiplex serology measurement and an additional 10 cases and matched control pairs from Greece were excluded due to unforeseen data restriction issues, resulting in a final sample set of 442 CRC cases and 442 controls for the present analysis.

### Selection and recombinant expression of E. coli and ETBF proteins

For the development of *E. coli* and ETBF multiplex serology, we specifically selected proteins based on their function in biofilm formation and toxicity/virulence of the respective bacterium (Table 1). Regarding *E. coli*, proteins relevant for attachment to host cells, aggregation and biofilm formation were selected. These included three autotransporters (Adhesin involved in diffuse adherence (AIDA-I), antigen 43 (Ag43), and TibA), proteins of the Type I pilus (FimA and FimH), and protein CsgA, a component of curli, the major protein constituent of the biofilm matrix.^28,29^ The proteins were recombinantly expressed without their signal peptide to enhance solubility. Moreover, for AIDA-I, Ag43, and TibA, only the N-terminal adhesin domain was expressed since the full-length protein would have exceeded the maximum size of 100 kDa for efficient recombinant bacterial expression, and the C-terminal domain contained multiple-transmembrane domains that would have diminished solubility.^30^ To potentially detect antibodies specific for the pks+ *E. coli* strain we further selected protein ClbM, an MATE family efflux transporter, expressed from the *pks+* island and responsible for translocation of the *E. coli* toxin colibactin.^11,31^ To serologically detect ETBF we specifically included two isoforms of the *B. fragilis* toxin (BFT-1 and BFT-2) since these were reported to be the most abundant isoforms in CRC tissue samples.^13^ For both proteins, the amino acid sequence representing the mature toxin was selected for recombinant expression. All proteins were recombinantly expressed as GST-tagged fusion proteins in *E. coli* BL21, as described previously.^32^

**Table 1.** Selected *E. coli* and ETBF proteins for multiplex serology

					Cutoff [MFI]	
Species	Antigen	Uniprot ID	Selected AA	Predicted Function	α-IgA	α-IgG
*E. coli*	FimA	P04128	24–182	Fimbriae major subunit	100	100
	FimH	P08191	22–300	Fimbriae minor subunit	100	250
	CsgA	P28307	21–151	biofilm structure Curli	100	300
	Ag43	P39180	53–551	Adhesin domain of autotransporter	400	600
	AIDA-I	Q03155	50–846	Adhesin domain of autotransporter	250	650
	TibA	Q9XD84	55–677	Adhesin domain of autotransporter	150	200
	ClbM	Q0P7K3	full length	Colibactin MATE family efflux transporter	100	150
ETBF	BFT-1	Q9S5W0	212–397	ETBF toxin isoform 1	150	300
	BFT-2	O05091	212–397	ETBF toxin isoform 2	100	250

AA, Amino acids; ETBF, Enterotoxigenic *Bacteroides fragilis*; MFI, Median fluorescence intensity

### Multiplex serology

The GST-tagged fusion proteins described above were affinity-purified on glutathione-casein coated fluorescently labeled polystyrene beads (Luminex Corp., Austin, TX, USA) as previously described.^32^ High-throughput simultaneous analysis of several antigens per serum sample was enabled by mixing the bead sets loaded with distinct antigens.

Sera were pre-incubated in a 1:50 dilution in a buffer containing polyvinylalcohol, polyvinylpyrrolidon and 1 g/l casein to suppress nonspecific binding of antibodies to the glutathione-coated beads and 0.33 g/l protein lysate of *E. coli* over-expressing GST-tag. The latter was titrated to block unspecific signals against the GST-tag sequence and any residual bacterial proteins while retaining specific signals to the recombinantly expressed proteins. After the pre-incubation step, sera were incubated with the antigen-loaded bead mixture and bound IgG or IgA serum antibodies were labeled separately by biotinylated secondary antibodies (goat anti-Human IgG-Biotin #109-065-098 and goat anti-Human IgA-Biotin #109-065-011, Jackson ImmunoResearch, Westgrove, PA, USA) and a subsequent incubation with Streptavidin-R-Phycoerythrin (MossBio, Pasadena, MD, USA). A Luminex 200 Analyzer (Luminex Corp., Austin, TX, USA) was then used to distinguish the bead sets and their respective antigens and to quantify the amount of serum IgG or IgA bound to the antigen. The level of antibody response was given as the median fluorescence intensity (MFI) of at least 100 beads per type measured. Background values against the GST-tag, as well as the bead-surface and secondary reagents were subtracted to generate net MFI values.

Antigen-specific cutoffs (Table 1) were defined as described previously at the approximate inflection point of frequency distribution curves under the assumption that a sudden rise in the distribution of antibody response over the percentile of sera indicates a cutoff for sero-positivity (i.e., MFI values were plotted against the percentage of sera that had at least that MFI. The cutoff was then set where a higher cutoff would not significantly alter the sero-positivity rate).^33,34^ The overall sero-positivity to each bacterium was arbitrarily defined as being positive for any of the recombinantly expressed proteins per bacterium since no gold standard serology test was available to validate the newly developed assay.

### Statistical analysis

Continuous IgA and IgG antibody responses to *E.coli* and ETBF proteins (in MFI) were compared using a Wilcoxon Mann–Whitney test among sero-positive individuals only since antibody responses to some of the antigens were mostly below the technical limit of the assay (100 MFI).

In the crude model, univariate conditional logistic regression was used to estimate odds ratios (ORs), and the corresponding 95% confidence intervals (CIs), for the association of antibody responses to individual *E. coli* and ETBF proteins and positivity to any protein in either bacterium. As *E. coli* and ETBF were previously reported together in biofilms associated with CRC tumor tissue,^21^ we assessed combinations of these microbes (*E. coli* and ETBF negative, *E. coli* or ETBF positive, *E. coli* and ETBF positive) with the odds of developing CRC.

In a first multivariable adjustment, the following covariates were included: body mass index (BMI, kg/m2, continuous variable), smoking status (never, former, current), alcohol consumption (g/d, continuous variable), and highest education attained at baseline (≤primary school, technical/professional, ≥secondary school). Missing values in the respective variables were handled as their own category. We further performed a second multivariable adjustment with additional consideration of dietary variables (total daily intake in [g/d] of vegetables, fruits, dairy, cereals, fish, red meats, processed meats, fiber, and daily intake level of total energy [kcal], all continuous) and physical activity (inactive, moderately inactive, moderately active, active). No violations of the assumption of linearity were observed for continuous variables, as assessed by visual inspection of the data, by comparing the findings from models where the variables were integrated as continuous versus models where the variables were categorized and by confirming that the odds for the association of each adjustment variable with CRC was linear when the variable was modeled as categorical.

Previously published literature^22,23^ has suggested that biofilms are predominantly found in proximal rather than in distal colon cancer. Therefore, we assessed the association of *E. coli* and ETBF serology with cancer separately in the proximal and distal colon. A *p*-value for heterogeneity was determined to assess a statistically significant difference in the association between the two sites. No null hypothesis tests were used to assess associations with unknown subsites (n = 44) or rectal cancer (n = 51) because the frequencies were too sparse.

As a sensitivity analysis, we repeated the above-described analysis excluding all cases with a blood draw ≤2 y before diagnosis as well as their respective matched controls.

All statistical analyses were performed with SAS version 9.4 (SAS Institute, Cary, NC, USA). A two-sided *p*-value below 0.05 was considered statistically significant. Multiple-testing adjustment for tests on antigen combinations was conducted with the False Discovery Rate (FDR) correction using the SAS Multitest procedure.

## Results

### Characteristics of the study population

The mean age in our study population was 59 y (standard deviation 8 y) and 51% of the participants were women. The median time between blood draw and diagnosis was 3.4 y and ranged between 0 and 8.5 y. Cases tended to be more likely obese (BMI ≥30 kg/m^2^) than controls (21% versus 16%, respectively), to be a former or current smoker (59% versus 52%, respectively) and to have a higher daily energy intake (mean: 2173 kcal versus 2052 kcal, respectively) (Table 2).

**Table 2.** Selected baseline characteristics of incident colorectal cancer cases and controls, the European Prospective Investigation into Cancer and Nutrition (EPIC) study

	Controls (n = 442)	Cases (n = 442)
Sex, n (%)^a^		
Women	225 (51)	225 (51)
Men	217 (49)	217 (49)
Age at blood draw [y], mean (SD) ^a^	59 (8)	59 (8)
Country, n (%)^a^		
France	10 (2)	10 (2)
Italy	93 (21)	93 (21)
Spain	74 (17)	74 (17)
United Kingdom	127 (29)	127 (29)
The Netherlands	67 (15)	67 (15)
Germany	71 (16)	71 (16)
Education, n (%)		
≤Primary school	186 (43)	192 (46)
Technical/professional	110 (26)	87 (21)
≥Secondary school	132 (31)	142 (34)
Missing	14	21
BMI [kg/m^2^], n (%)		
<25	155 (35)	149 (34)
25–29.9	215 (49)	199 (45)
≥30	72 (16)	94 (21)
Smoking status, n (%)		
Never	209 (48)	180 (41)
Former	140 (32)	170 (39)
Current	91 (20)	88 (20)
Missing	2	4
Alcohol intake at baseline^b^ [g/d], n (%)		
<6	212 (48)	190 (43)
6–20	117 (26)	121 (27)
>20	113 (26)	130 (29)
Physical activity^c^, n (%)		
Inactive	139 (32)	149 (34)
Moderately inactive	128 (29)	145 (33)
Moderately active	83 (19)	78 (18)
Active	88 (20)	66 (15)
Missing	4	4
Daily dietary intake, mean (SD)		
Total energy [kcal]	2052 (616)	2173 (839)
Total vegetables [g]	195 (121)	190 (125)
Total fruits [g]	255 (185)	257 (203)
Dairy [g]	339 (232)	315 (251)
Cereals [g]	225 (148)	220 (123)
Fiber [g]	23 (8)	23 (9)
Fish [g]	34 (35)	33 (33)
Red meats [g]	42 (29)	46 (40)
Processed meats [g]	30 (28)	37 (66)

BMI, body mass index; g, grams; kcal, kilocalories; SD, standard deviation; ^a^Cases and controls were matched by incidence-density sampling on these variables; ^b^Data missing for one of the cases; ^c^Cambridge physical activity index; The numbers of each category may not add up to the total number of cases and controls because of missing values.

### Antibody responses to E. coli and ETBF proteins in CRC cases and controls

**Figure 1.**
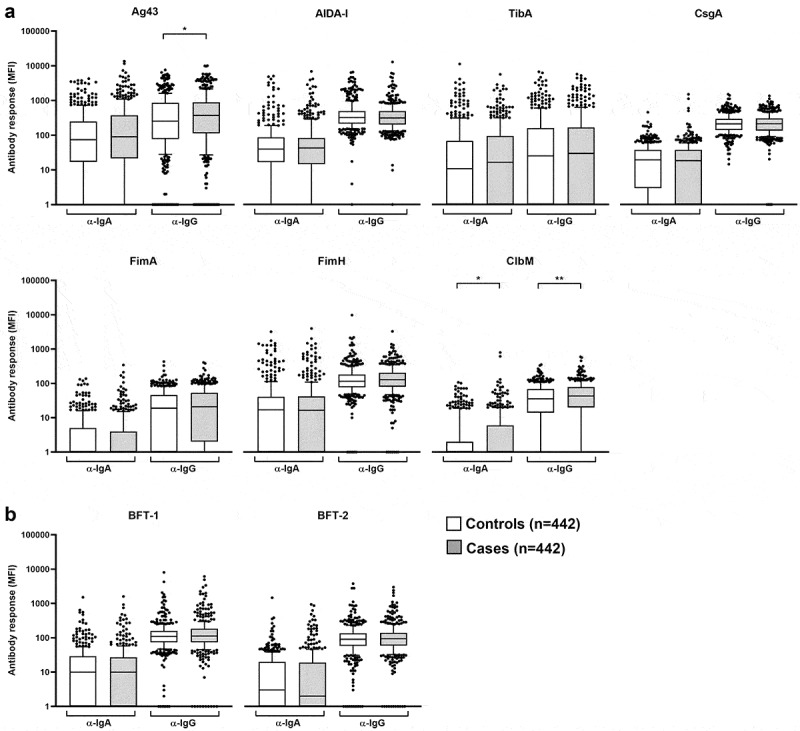
IgA and IgG antibody responses (median fluorescence intensities, MFI) to (a) *E. coli* and (b) ETBF proteins in cases and controls in the EPIC study. Boxes represent 25^th^ to 75^th^ and whiskers the 5^th^ to 95^th^ percentiles, solid lines show the median. Dots represent data points lying outside the 5^th^ and 95^th^ percentiles, respectively. Wilcoxon Mann–Whitney test was applied to compare continuous antibody responses [MFI] between sero-positive controls and cases

We separately measured IgA and IgG antibody responses to *E. coli* and ETBF proteins and compared the level of response, given by continuous MFI, between cases and controls (Figure 1). This analysis was performed among sero-positive cases and controls only since antibody levels for some of the antigens were mostly below the technical limit of the assay (100 MFI). No statistically significant difference was observed in antibody level between cases and controls. In this study, we consequently decided not to analyze the antibody response levels in more detail but rather focus on sero-positivity as a more robust measure.

### Association of sero-positivity to E. coli and ETBF proteins with odds of developing CRC

Applying cutoffs for sero-positivity (Table 1) defined 42% of the controls as IgA-positive to any *E. coli* protein compared to 49% of the cases (Table 3). This difference was statistically significant with an OR of 1.37 for CRC in the crude model (95% CI: 1.04, 1.80; *p* value = 0.024), 1.35 in the multivariable-adjusted model 1 (95% CI: 1.02, 1.79; *p*-value = 0.035), and 1.42 in the multivariable-adjusted model 2 adjusting additionally for dietary variables and physical activity (95% CI: 1.05, 1.91; *p*-value = 0.022). However, the FDR-adjusted values did not retain significance with corresponding q-values of 0.096, 0.118 and 0.088, respectively. Given that the *p*-values are derived from a clear hypothesis-driven approach with a small number of comparisons across two bacteria, we base our interpretation on the observed *p*-values and present the q-values as a conservative balance.

**Table 3.** Sero-positivity to *E. coli* and ETBF proteins and association with odds of developing colorectal cancer, the EPIC study

			Positive n (%)		Crude Model		Multivariable Model 1		Multivariable Model 2	
Secondary antibody	Species	Antigen	Controls n = 442	Cases n = 442	OR (95% CI)^b^	*p-value^b^*	OR (95% CI)^c^	*p-value^c^*	OR (95% CI)^d^	*p-value^d^*
α-IgA	*E. coli*	Ag43	72 (16)	103 (23)	**1.53 (1.10, 2.12)**	***0.012***	**1.56 (1.11, 2.19)**	***0.011***	**1.55 (1.08, 2.21)**	***0.018***
		AIDA-I	38 (9)	50 (11)	1.34 (0.87, 2.08)	*0.187*	1.34 (0.85, 2.12)	*0.207*	1.46 (0.90, 2.35)	*0.125*
		TibA	74 (17)	83 (19)	1.15 (0.81, 1.63)	*0.425*	1.13 (0.79, 1.62)	*0.508*	1.18 (0.81, 1.73)	*0.398*
		FimA	3 (1)	5 (1)	1.67 (0.40, 6.97)	*0.484*	1.61 (0.37, 6.96)	*0.527*	2.24 (0.50, 9.93)	*0.290*
		FimH	49 (11)	48 (11)	0.98 (0.64, 1.49)	*0.915*	0.97 (0.63, 1.50)	*0.902*	1.05 (0.66, 1.66)	*0.841*
		CsgA	14 (3)	16 (4)	1.14 (0.56, 2.34)	*0.715*	1.30 (0.62, 2.74)	*0.489*	1.26 (0.58, 2.76)	*0.563*
		ClbM	2 (0)	5 (1)	2.50 (0.49, 12.89)	*0.273*	2.83 (0.53, 15.07)	*0.223*	3.26 (0.56, 18.87)	*0.187*
		Any	184 (42)	217 (49)	**1.37 (1.04, 1.80)**	***0.024***	**1.36 (1.03, 1.81)**	***0.031***	**1.42 (1.05, 1.92)**	***0.021***
	ETBF	BFT-1	16 (4)	16 (4)	1.00 (0.50, 2.00)	*1.000*	1.06 (0.52, 2.16)	*0.881*	1.05 (0.50, 2.22)	*0.894*
		BFT-2	11 (2)	22 (5)	2.00 (0.97, 4.12)	*0.061*	1.88 (0.90, 3.94)	*0.095*	1.89 (0.87, 4.11)	*0.108*
		Any	23 (5)	27 (6)	1.17 (0.67, 2.05)	*0.572*	1.15 (0.65, 2.03)	*0.631*	1.16 (0.64, 2.11)	*0.619*
	Neither *E. coli*^a^ nor ETBF^a^		247 (56)	218 (49)	1.00 (ref)		1.00 (ref)		1.00 (ref)	
	*E. coli*^a^ or ETBF^a^		183 (41)	204 (46)	1.29 (0.97, 1.70)	*0.076*	1.28 (0.96, 1.71)	*0.090*	1.35 (1.00, 1.83)	*0.051*
	*E. coli*^a^ and ETBF^a^		12 (3)	20 (5)	1.91 (0.92, 3.97)	*0.084*	1.84 (0.87, 3.09)	*0.109*	1.82 (0.83, 3.99)	*0.137*
α-IgG	*E. coli*	Ag43	139 (31)	148 (33)	1.10 (0.83, 1.47)	*0.511*	1.17 (0.87, 1.57)	*0.309*	1.17 (0.86, 1.60)	*0.308*
		AIDA-I	84 (19)	73 (17)	0.85 (0.60, 1.19)	*0.337*	0.87 (0.61, 1.24)	*0.434*	0.87 (0.60, 1.27)	*0.477*
		TibA	102 (23)	102 (23)	1.00 (0.73, 1.38)	*1.000*	1.03 (0.74, 1.44)	*0.851*	1.06 (0.75, 1.51)	*0.727*
		FimA	27 (6)	37 (8)	1.39 (0.84, 2.29)	*0.206*	1.47 (0.87, 2.49)	*0.149*	1.46 (0.84, 2.52)	*0.180*
		FimH	65 (15)	76 (17)	1.22 (0.84, 1.79)	*0.293*	1.19 (0.81, 1.76)	*0.382*	1.27 (0.85, 1.92)	*0.247*
		CsgA	119 (27)	138 (31)	1.26 (0.93, 1.71)	*0.142*	1.28 (0.93, 1.76)	*0.131*	1.27 (0.91, 1.77)	*0.168*
		ClbM	13 (3)	20 (5)	1.58 (0.77, 3.26)	*0.213*	1.60 (0.76, 3.39)	*0.218*	1.72 (0.79, 3.74)	*0.171*
		Any	283 (64)	307 (69)	1.28 (0.96, 1.69)	*0.089*	1.33 (0.99, 1.78)	*0.057*	1.29 (0.95, 1.75)	*0.105*
	ETBF	BFT-1	28 (6)	43 (10)	1.58 (0.97, 2.68)	*0.069*	**1.70 (1.02, 2.83)**	***0.040***	**1.80 (1.06, 3.04)**	***0.029***
		BFT-2	38 (9)	43 (10)	1.14 (0.73, 1.78)	*0.569*	1.23 (0.77, 1.96)	*0.383*	1.28 (0.79, 2.07)	*0.323*
		Any	49 (11)	64 (14)	1.35 (0.91, 2.00)	*0.137*	1.45 (0.96, 2.18)	*0.076*	1.45 (0.95, 2.22)	*0.085*
	Neither *E. coli*^a^ nor ETBF^a^		144 (33)	123 (28)	1.00 (ref)		1.00 (ref)		1.00 (ref)	
	*E. coli*^a^ or ETBF^a^		264 (60)	267 (60)	1.17 (0.87, 1.57)	*0.303*	1.24 (0.91, 1.69)	*0.167*	1.22 (0.89, 1.68)	*0.212*
	*E. coli*^a^ and ETBF^a^		34 (8)	52 (12)	**1.74 (1.07, 2.82)**	***0.026***	**1.87 (1.13, 3.09)**	***0.015***	**1.79 (1.07, 3.00)**	***0.028***

^a^Positive to any protein for the respective species; ^b^Crude Model: Conditional logistic regression model based only on the matching factors;

^c^Multivariable model 1: crude model plus additional adjustment for BMI (kg/m^2,^ continuous), smoking status (never, former, current), alcohol consumption (g/d, continuous), highest education attained at baseline (≤primary school, technical/professional, ≥secondary school);

^d^Multivariable model 2: Multivariable model 1 plus additional adjustment for dietary variables (total daily intake in [g] of vegetables, fruits, dairy, cereals, fish, red meats, processed meats, fiber, and daily intake level of total energy [kcal], all continuous) and physical activity (inactive, moderately inactive, moderately active, active);

statistically significant associations (*p* < 0.05) are marked in bold font.

There was no statistically significant difference observed for being IgG-positive to any *E. coli* protein (controls: 64%, cases 69%) or being IgA- or IgG-positive to any BFT isoform (IgA: controls, 5%, cases 6%; IgG: controls, 11%, cases 14%). Among the individual *E. coli* proteins assessed, sero-prevalence in controls was lowest for ClbM (IgA: 0% and IgG: 3%) and highest for Ag43 (IgA: 16% and IgG: 31%). For ETBF, sero-prevalence was similarly low for both isoforms (BFT-1: IgA, 4% and IgG: 6%; BFT-2: IgA: 2% and IgG: 9%). Only IgA positivity to *E. coli* protein Ag43 (OR: 1.55; 95% CI: 1.08, 2.21) and IgG positivity to ETBF toxin BFT-1 (OR: 1.80; 95% CI: 1.06, 3.04) were significantly associated with higher odds of developing CRC.

To address the hypothesis of a combination of sero-positivity to both bacteria being associated with CRC, we examined positivity to any *E. coli* and/or ETBF protein, for IgA and IgG, respectively (Table 3). A dual-positive IgG immune response to both bacteria was more common in cases (12%) than controls (8%) resulting in a statistically significant 1.79-fold higher odds of developing CRC (95% CI: 1.07, 3.00; *p*-value = 0.028), with a corresponding q-value of 0.228. A similar point estimate was observed for IgA dual-positivity (OR: 1.82; 95% CI: 0.83, 3.99); however, this was not statistically significant and only included 5% of the cases and 3% of the controls.

We further assessed whether IgA or IgG positivity to any *E. coli* or ETBF protein or a combination thereof, was more strongly associated with higher odds of developing cancer at the major anatomical proximal or distal subsites of colon cancer (Table 4). Both IgA and IgG positivity to any *E. coli* protein, any ETBF protein, and dual-positivity to both was more common in proximal than distal colon cancer (Table 4). Moreover, IgG positivity to any *E. coli* protein, to either *E. coli* or ETBF and dual IgG-positivity to *E. coli* and ETBF were statistically significantly associated with an increased odds of developing proximal colon cancer (OR: 2.12; 95% CI: 1.20, 3.76, OR: 1.87; 95% CI: 1.04, 3.34, OR: 2.75; 95% CI: 1.16, 6.50, respectively) but not distal colon cancer. However, *p*-values for heterogeneity do not indicate a statistically significant difference between sites (0.093 and 0.095, respectively).

**Table 4.** Sero-positivity to *E. coli* and ETBF proteins and association with the odds of developing proximal or distal colon cancer, the EPIC study

		Proximal colon			Distal colon		
		Positive n (%)			Positive n (%)		
		Controls	Cases		Controls	Cases	
Secondary antibody		n = 154	n = 154	OR (95% CI)^b^	n = 193	n = 193	OR (95% CI)^b^
α-IgA	*E. coli*^a^	67 (44)	81 (53)	1.49 (0.87, 2.53)	81 (42)	92 (48)	1.34 (0.82, 2.17)
	ETBF^a^	10 (6)	14 (9)	1.64 (0.62, 4.34)	11 (6)	10 (5)	0.78 (0.30, 2.03)
	Neither *E. coli*^a^ nor ETBF^a^	83 (54)	69 (45)	1.00 (ref)	106 (55)	99 (51)	1.00 (ref)
	*E. coli*^a^ or ETBF^a^	65 (42)	75 (49)	1.52 (0.89, 2.61)	82 (42)	86 (45)	1.17 (0.72, 1.90)
	*E. coli*^a^ plus ETBF^a^	6 (4)	10 (6)	1.90 (0.55, 6.54)	5 (3)	8 (4)	1.52 (0.43, 5.34)
α-IgG	*E. coli*^a^	93 (60)	112 (73)	**2.12 (1.20, 3.76)**^C^	123 (64)	128 (66)	1.07 (0.67, 1.71)
	ETBF^a^	20 (13)	29 (19)	1.53 (0.77, 3.02)	18 (9)	21 (11)	1.21 (0.56, 2.61)
	Neither *E. coli*^a^ nor ETBF^a^	55 (36)	38 (25)	1.00 (ref)^d^	65 (34)	61 (32)	1.00 (ref)
	*E. coli*^a^ or ETBF^a^	85 (55)	91 (59)	**1.87 (1.04, 3.34)**	115 (60)	115 (60)	0.99 (0.60, 1.62)
	*E. coli*^a^ plus ETBF^a^	14 (9)	25 (16)	**2.75 (1.16, 6.50)**	13 (7)	17 (9)	1.37 (0.57, 3.33)

^a^Positive to any protein for the respective species;

^b^Conditional logistic regression model based on the matching factors plus additional adjustment for BMI (kg/m^2,^ continuous), smoking status (never, former, current), alcohol consumption (g/d, continuous), highest education attained at baseline (≤primary school, technical/professional, ≥secondary school), dietary variables (total daily intake in [g] of vegetables, fruits, dairy, cereals, fish, red meats, processed meats, fiber, and daily intake level of total energy [kcal], all continuous) and physical activity (inactive, moderately inactive, moderately active, active); statistically significant associations (*p* < 0.05) are marked in bold font;

^c^P-value for heterogeneity by anatomical sub-site = 0.093; ^d^P-value for heterogeneity by anatomical sub-site = 0.095.

A sensitivity analysis excluding cases diagnosed within 2 y after blood draw did not identify substantial differences in the observed associations (**Supplementary table S1**). IgA/IgG dual-positivity to *E. coli* gave an equivalent estimate of higher odds of developing CRC (OR: 1.57; 95% CI: 1.07, 2.32) as for IgA alone, while the combined positivity to both bacteria showed similar (though non-significant) associations with CRC to the individual Ig class. However, these analyses were limited in scope due to the few numbers of dual positives to ETBF (**Supplementary table S2**).

## Discussion

In the present CRC case–control study nested within a European prospective cohort, we observed that antibody positivity to *E. coli* proteins involved in biofilm formation and colibactin secretion, and to the ETBF toxin were associated with higher odds of developing CRC. Specifically, IgG dual-positivity to *E. coli* and ETBF was associated with a 1.79-fold higher odds overall, and a 2.75-fold higher odds of developing cancer in the proximal colon. While these findings did not retain significance after FDR multiple-testing correction, we contend that such corrections are over-stringent, as all our analyses were planned a priori, based on our stated hypothesis, with a modest number of related tested antigens.

To our knowledge, this is the first serological study assessing the association of *E. coli* and ETBF with the odds of developing CRC. Previous studies focused on the detection of these bacteria directly in tumor tissue or stool samples. Our findings are concordant with the landmark study by Dejea et al. (2018), which described the presence of biofilms consisting of both pks+ *E. coli* and ETBF in tumor tissue of CRC patients with FAP.^21^ In our study, information on this inherited condition is unavailable and it is not likely to be present in more than a few cases.^21^ Nevertheless, the presence of biofilms in colonic tissue has been demonstrated by studies of sporadic CRC patients, particularly in tumors arising from the proximal colon.^22,23^ Overall, 49% of the cases in our study were IgA-positive and 69% of the cases were IgG-positive to any of the seven *E. coli* proteins included in the assay. Although we are not aware of previous serological studies for which we can compare the sero-prevalence obtained in this study, several studies have detected the respective bacteria directly in tissue. Studies ascertaining pks+ *E. coli* in tumor tissue reported a lower prevalence of 22% to 55% in CRC cases.^14–16^ Our serological assay, however, included proteins that are not necessarily pks+ *E. coli* strain specific but also generally relevant for attachment to host cells, aggregation, and biofilm formation. These comprised autotransporters AIDA-I, Ag43, TibA, proteins of the Type I pilus FimA and FimH, and protein CsgA, a component of curli, the major protein constituent of the biofilm matrix.^28,29^ Sero-prevalence for these proteins among cases varied with the lowest prevalence observed for FimA (IgA 1% and IgG 8%) and highest for Ag43 (IgA 23% and IgG 33%). This difference in prevalence was even apparent for proteins of the same organelle, i.e., FimA and FimH as part of the type I pilus. This could be likely due to either the immunogenicity of the recombinantly expressed proteins and/or how well these proteins are recognized by the host immune system. IgA sero-positivity to Ag43 was the sole antigen singly significantly associated with higher odds of developing CRC in our study, which may indicate the importance of this autotransporter in CRC development. Specifically, for pks+ *E. coli*, there was a tumor mutational signature identified by Pleguezuelos-Manzano et al. (2020) in approximately 5% of CRC cases from two independent cohorts.^19^ This frequency was concordant with the sero-prevalence to protein ClbM (IgA: 1%, IgG: 5%), a MATE family efflux transporter expressed from the *pks+* island and responsible for translocation of the *E. coli* toxin colibactin.^11,31^ Studies on the prevalence of ETBF in CRC tissue have reported rates ranging from 26% to 68%.^13,16,17^ Similarly, a study analyzing fecal samples found 32% of CRC patients positive for ETBF DNA.^35^ Although we included two isoforms of BFT, BFT-1 and BFT-2, to potentially increase the sensitivity of the serology assay, a lower overall BFT prevalence was observed (IgA: 6%, IgG: 14%). Similarly, dual sero-positivity to *E. coli* and ETBF was lower in CRC cases of our study (IgA: 5%, IgG: 12%) than reported in Dejea et al. (2018), albeit for CRC patients with FAP (52%).^21^ One must consider that serology is an indirect and systemic measure of infections. We attempted to detect mucosal and systemic antibody responses by respective, separate detection of IgA and IgG, however, these do not necessarily need to originate from infections in the colorectum. Moreover, a serological gold standard assay was not available to validate our newly developed assays, and thus, recombinantly expressed proteins might lack immunogenicity. Altogether, these components may partly explain differences in the sero-prevalence of the bacteria obtained in our study compared to previous reports directly detecting the bacterial species in tumor tissue or stool samples from CRC patients. A recent study by Messaritakis et al. (2020) directly detected bacterial DNA in the blood of healthy donors and CRC patients assessing bacterial translocation of the gut epithelial barrier.^36^ The authors did not further determine the infecting *E. coli* or *B. fragilis* strain but found *E. coli* DNA in 16% of the healthy donors and 26% of the cases as well as *B. fragilis* DNA in 0% and 55%, respectively. A direct comparison of these data with our serological assays would further contribute to a better understanding of the findings obtained in this study.

The ORs for IgA- and IgG-positivity in the association with CRC were similar in our study, although for *E. coli* and ETBF dual-positivity they were statistically significant only for measurement of IgG. This observation may simply result from the higher sero-prevalence of IgG antibodies or chance findings from the low prevalence of subjects with dual-positivity. Overall, quantitative IgA antibody responses to all included proteins were lower than the respective IgG ones. This was also reflected in the resulting sero-prevalence after application of antigen- and Ig class-specific cutoffs for the MFI values. However, we are also unaware of the timing of infection relative to tumorigenesis as well as the natural history of antibody responses to the two bacterial species. A sensitivity analysis excluding cases diagnosed within 2 y after blood draw did not diminish observed associations. It remains, however, unclear to what extent these microbes may infect healthy colon tissue prior to carcinogenesis or whether they only infect a developing tumor due to bacterial translocation across the impaired gut-barrier integrity. In both scenarios, the bacteria may induce mucosal antibody responses of the IgA and/or systemic antibody responses of the IgG class and potentially exert pro-carcinogenic effects. A secondary analysis considering IgA/IgG dual-positivity to either *E. coli* or ETBF or both species, provided similar estimates for the odds of developing CRC as for positivity of the individual Ig class alone but need to be considered cautiously as there were few cases or controls dual positive to ETBF. Since we lack both the ability to estimate the time-point of seroconversion in our study and information on the presence of precancerous lesions at the time of blood draw, our data do not provide answers to these questions. An illustrative example from the literature describes the bacterial driver-passenger model. In this, the observed association of the bacterium *Streptococcus gallolyticus* with CRC development is hypothesized to result from a ‘passenger bacterium’ effect causing a local and systemic infection after the colon epithelial barrier has been impaired due to tumor formation.^37^ The ETBF, in contrast, is hypothesized to be a ‘driver bacterium’ with the ability to initiate CRC development due to its pro-carcinogenic effects.^37^ The mechanism of infection and biofilm formation in colon tissue by *E. coli* and ETBF, the resulting host immune response, and the potential carcinogenic properties of the two bacteria may therefore be an early event in colorectal carcinogenesis. However, this hypothesis warrants further studies to better understand the potential implications for the pathogenic sequence in CRC development.

Our study has several notable limitations and strengths. First, the selection of proteins for the multiplex serology assay was not based on an unbiased approach but focused on previously published literature on potentially relevant protein function. Thus, little was known about the immunogenicity of these proteins prior to our study. To our knowledge, there was no serological gold standard assay available for validation and our results from using this novel approach require confirmation in independent studies. A major advantage of using serology compared to tumor tissue-based assays is that most cohorts store blood samples of their study participants allowing for prospective analysis of associations with cancer risk. Moreover, the applied multiplex serology allows for high-throughput analyses of a large number of samples for several antigens as well as different Ig classes. This enabled us to directly compare the host antibody response to multiple bacterial species at once and the assessment of a more detailed immune response to the bacterium beyond just overall sero-positivity. Due to the assessment of numerous antigens and Ig classes, however, it may also be argued that correction of our findings for multiple comparisons is required. We wish to acknowledge that application for multiple testing would have removed significance of our results although this may be considered over-stringent due to the correlated nature of the tested antigens. Larger studies with more statistical power are therefore needed to confirm our findings. Generally, another strength of multiplex serology is the ability to quantify the level of antibody response. Due to the low immune response below the technical limit for some antigens, we compared quantitative antibody levels among sero-positives only and did not identify any antigen with statistically significantly different antibody levels between cases and controls. We instead focused on sero-positivity alone as a more robust measure for CRC association. As another limitation, we did not have the necessary data to assess the association of *E. coli* and ETBF sero-positivity with CRC by molecular status of the tumor (e.g., MSI or *APC* and *KRAS* mutation status). Assessments of association of the antibody responses with survival of the patients were also not conducted due to the limited available follow-up data post diagnosis. These remain interesting questions that should be addressed in future studies.^18^ Finally, residual confounding cannot completely be ruled out, despite controlling for relevant covariates. A major strength of the study design, however, is that it is based on a large multicenter cohort covering most of Western Europe with detailed prospective data collection and serum samples taken several years prior to diagnosis.

In conclusion, in this case–control study nested within EPIC, antibody responses to *E. coli* proteins and the ETBF toxin were associated with higher odds of developing CRC, predominantly of proximal tumor location. This first-time application of a newly developed *E. coli* and ETBF multiplex serology assay needs verification in other settings to assess whether the presence of these two bacterial species may increase the risk of developing CRC.

## Supplementary Material

Supplemental Material

## Data sharing

For information on how to submit an application for gaining access to EPIC data and/or biospecimens, please follow the instructions at http://epic.iarc.fr/access/index.php
