# Supplementary material for: Association of Pre-diagnostic Antibody Responses to Escherichia coli and Bacteroides fragilis Toxin Proteins with Colorectal Cancer in a European Cohort
Source: Gut Microbes. 2021 Apr 20;13(1):1903825. doi: 10.1080/19490976.2021.1903825 (PMC8078709; doi:10.1080/19490976.2021.1903825)
Supplement: Supplemental Material [file KGMI_A_1903825_SM8595.docx]

**Supplementary table S1: Sero-positivity to *E. coli* and ETBF proteins and odds of developing colorectal cancer, in blood samples drawn >2 years before diagnosis, the EPIC study.**

|  |  | **All** | | | **Proximal colon** | | | **Distal colon** | | |
| --- | --- | --- | --- | --- | --- | --- | --- | --- | --- | --- |
|  |  | **Positive n (%)** | |  | **Positive n (%)** | |  | **Positive n (%)** | |  |
| **Secondary** |  | **Controls** | **Cases** |  | **Controls** | **Cases** |  | **Controls** | **Cases** |  |
| **antibody** |  | **n=328** | **n=328** | **OR (95% CI)^b^** | **n=118** | **n=118** | **OR (95% CI)^b^** | **n=141** | **n=141** | **OR (95% CI)^b^** |
| α-IgA | *E. coli*^a^ | 136 (41) | 164 (50) | **1.46 (1.04, 2.07)** | 53 (45) | 62 (53) | 1.39 (0.72, 2.70) | 58 (41) | 67 (48) | 1.44 (0.80, 2.60) |
|  | ETBF^a^ | 21 (6) | 23 (7) | 1.09 (0.58, 2.07) | 9 (8) | 12 (10) | 1.75 (0.54, 5.61) | 10 (7) | 8 (6) | 0.77 (0.27, 2.19) |
|  | Neither *E. coli*^a^ nor ETBF^a^ | 183 (56) | 158 (48) | 1.00 (ref) | 62 (53) | 53 (45) | 1.00 (ref) | 78 (55) | 72 (51) | 1.00 (ref) |
|  | *E. coli*^a^ or ETBF^a^ | 133 (41) | 153 (47) | 1.41 (0.99, 2.00) | 50 (42) | 56 (47) | 1.52 (0.77, 2.98) | 58 (41) | 63 (45) | 1.28 (0.71, 2.32) |
|  | *E. coli*^a^ plus ETBF^a^ | 12 (4) | 17 (5) | 1.60 (0.70, 3.66) | 6 (5) | 9 (8) | 1.71 (0.40, 7.31) | 5 (4) | 6 (4) | 1.39 (0.34, 5.57) |
| α-IgG | *E. coli*^a^ | 203 (62) | 234 (71) | **1.52 (1.07, 2.17)** | 68 (58) | 91 (77) | **4.45 (1.92, 10.32)** | 90 (64) | 93 (66) | 1.07 (0.62, 1.85) |
|  | ETBF^a^ | 38 (12) | 47 (14) | 1.34 (0.82, 2.19) | 14 (12) | 26 (22) | **2.60 (1.05, 6.43)** | 16 (11) | 10 (7) | 0.69 (0.27, 1.73) |
|  | Neither *E. coli*^a^ nor ETBF^a^ | 112 (34) | 87 (27) | 1.00 (ref) | 45 (38) | 25 (21) | 1.00 (ref) | 47 (33) | 46 (33) | 1.00 (ref) |
|  | *E. coli*^a^ or ETBF^a^ | 191 (58) | 201 (61) | 1.40 (0.96, 2.02) | 64 (54) | 69 (58) | **2.81 (1.25, 6.29)** | 82 (58) | 87 (62) | 1.04 (0.58, 1.83) |
|  | *E. coli*^a^ plus ETBF^a^ | 25 (8) | 40 (12) | **2.00 (1.09, 3.67)** | 9 (8) | 24 (20) | **11.05 (2.86, 42.70)** | 12 (9) | 8 (6) | 0.81 (0.28, 2.37) |

^a^positive to any protein for the respective species; ^b^Conditional logistic regression model based on the matching factors plus additional adjustment for BMI (kg/m^2^, continuous), smoking status (never, former, current), alcohol consumption (g/day, continuous), highest education attained at baseline (≤primary school, technical/professional, ≥secondary school), dietary variables (total daily intake in [g] of vegetables, fruits, dairy, cereals, fish, red meats, processed meats, fibre, and daily intake level of total energy [kcal], all continuous) and physical activity (inactive, moderately inactive, moderately active, active); statistically significant associations (p < 0.05) are marked in bold font.

**Supplementary table S2: IgA/IgG dual-positivity to *E. coli* and ETBF proteins and odds of developing colorectal cancer, the EPIC study.**

|  |  | **Positive n (%)** | |  |
| --- | --- | --- | --- | --- |
|  | **Secondary** | **Controls** | **Cases** |  |
| **Bacterium** | **antibody** | **n=442** | **n=442** | **OR (95% CI)^b^** |
| *E. coli*^a^ | Neither α-IgA nor α-IgG | 117 (26) | 101 (23) | 1.00 (ref) |
|  | α-IgA or α-IgG | 183 (41) | 158 (36) | 0.96 (0.67, 1.39) |
|  | α-IgA plus α-IgG | 142 (32) | 183 (36) | **1.57 (****1.07, 2.32)** |
| ETBF^a^ | Neither α-IgA nor α-IgG | 377 (85) | 362 (82) | 1.00 (ref) |
|  | α-IgA or α-IgG | 58 (13) | 69 (16) | 1.31 (0.87, 1.96) |
|  | α-IgA plus α-IgG | 7 (2) | 11 (2) | 1.62 (0.58, 4.51) |
| Neither *E. coli*^a^ nor ETBF^a^ | Neither α-IgA nor α-IgG | 108 (24) | 89 (20) | 1.00 (ref) |
| *E. coli*^a^ or ETBF^a^ | α-IgA or α-IgG | 330 (75) | 346 (78) | 1.28 (0.91, 1.82) |
| *E. coli*^a^ plus ETBF^a^ | α-IgA plus α-IgG | 4 (1) | 7 (2) | 1.67 (0.44, 6.37) |

^a^positive to any protein for the respective species; ^b^Conditional logistic regression model based on the matching factors plus additional adjustment for BMI (kg/m^2^, continuous), smoking status (never, former, current), alcohol consumption (g/day, continuous), highest education attained at baseline (≤primary school, technical/professional, ≥secondary school), dietary variables (total daily intake in [g] of vegetables, fruits, dairy, cereals, fish, red meats, processed meats, fibre, and daily intake level of total energy [kcal], all continuous) and physical activity (inactive, moderately inactive, moderately active, active); statistically significant associations (p < 0.05) are marked in bold font.
